# Fatigue effects on the knee flexors neuromuscular parameters during repeated sprinting

**DOI:** 10.1186/s13102-023-00789-y

**Published:** 2024-01-02

**Authors:** Ricardo Pimenta, Tomás Lopes, A. P. Veloso

**Affiliations:** 1 Faculdade de Motricidade Humana, CIPER, Universidade de Lisboa, Cruz Quebrada Dafundo, Lisboa, Portugal; 2 Research Center of the Polytechnic Institute of Maia (N2i), Maia Polytechnic Institute (IPMAIA), Castêlo da Maia, 4475-690 Maia, Portugal; 3 Futebol Clube Famalicão - Futebol SAD, Department of Rehabilitation and Performance, Famalicão, Portugal; 4 Department of Biochemistry, King’s College London, Oxford, UK

**Keywords:** Repeated sprints ability, Neuromuscular fatigue, Speed, Peak Torque, Rate of Torque Development, Hamstrings

## Abstract

**Background:**

To identify at which point fatigue on neuromuscular parameters occurs in the knee flexors during a repeated sprint protocol.

**Methods:**

Physical active males without previous hamstring strain injury were recruited. Neuromuscular parameters such as peak torque (PT) and rate of torque development (RTD) were assessed after every two sprints in a 5 × (2 x 30 m) repeated sprint protocol.

**Results:**

Twenty physical active males participated in the study. A significant effect of sprint number was found (*p* < 0.001; η^2^_p_ = 0.643) with a decreased sprint speed by 6.9% from fastest to slowest sprint. No significant differences were observed in the time between finishing the sprint and performing the first MVIC (46.3 ± 4.7s; *p* = 0.423), nor in the time between finishing a set and starting the next set (121.2 ± 7.6s; *p* = 0.503). Regarding neuromuscular parameters, the only significant difference found was in PT between before and after two sprints (117.95 ± 5.61 N⋅m vs. 110.64 ± 5.71 N⋅m; *p* = 0.048, *d* = 0.289) and on RTD 0-50ms before and after ten sprints (465.78 ± 223.76 N⋅m/s vs. 382.30 ± 189.56 N⋅m/s; *p* = 0.008; η^2^_p_ = 0.149).

**Conclusions:**

A recovery time of 46s between sprints and testing neuromuscular parameters (due to experimental design) seems sufficient to restore the neuromuscular system. Therefore, it can be suggested that time recovery is the principal factor in detecting fatigue on neuromuscular parameters.

## Introduction

The capacity to perform high-intensity sprinting multiple times is a key performance factor in team sports which is often evaluated by repeated-sprint ability (RSA) tests [1] and depends on metabolic, neural, and mechanical factors [1]. Indeed, RSA has been characterized as the ability to produce the best possible average sprint performance over a series of sprints (≤ 10 s), separated by short (≤ 60 s) recovery periods [1]. Repeated sprint performance testing has been inconsistent between studies due to different exercise-to-rest ratios which differently alter the neuromuscular and metabolic systems [2–4]. Indeed, significant performance decrements in cycling sprint performance after four [2] and six sprints [3] and in running sprint performance after the fifth sprint [4] have been reported.

The decrease in running sprints performance can be related to changes in the neuromuscular system caused by muscle fatigue, which can be evaluated using parameters such as peak torque (PT) [5] and rate of torque development (RTD). PT is usually considered an indicator of peripheral fatigue which is characterized by morphological features such as changes in the cross-sectional area since a larger physiological cross-sectional area of the biceps femoris long head was related to attenuated hamstring strength loss immediately after a 15 × 30 m intermittent sprint protocol [5]. Furthermore, during the protocol, fatigue could also impact the RTD [6], which is strongly influenced by the neural drive specifically to the discharge and recruitment of the motor units [7, 8].

In sprint performance, the ability to produce torque during the first milliseconds is considered a critical factor when fast and/or forceful muscle contractions are required [9]. Therefore, for sprinting, this ability is paramount and corresponds to pushing onto the ground in the posterior direction by quickly generating a large hip extensor moment using the hamstring muscles [10]. The hamstrings are the main knee flexors and hip extensors, being the most affected by strain injuries in sports that involve running and jumping, with sprinting as the most common mechanism [11–13]. The hamstrings are more fatigable than the quadriceps [14], and have a higher injury rate [15]. However, most research conducted on knee flexor muscle fatigue also measured quadriceps fatigue simultaneously, generally through hamstring:quadriceps ratios [16, 17], measuring knee flexor muscle fatigue in different methodological conditions and one limb at a time. Given the short window to detect fatigue, simultaneous measurement of both limbs allows to more accurately detect fatigue effects between limbs, which could be crucial to compare injured and non-injured limbs. It should be noted that despite the volume of studies analyzing repeated sprints [18] the specific volume of running sprints and rest periods necessary to induce fatigue on neuromuscular parameters (PT and RTD) in the knee flexors is unclear. This information would allow researchers to minimize data collection and processing, as well as to reduce unnecessary athlete exposure to sprints tests which could be relevant to future studies when the goal is to study knee flexors fatigue. Therefore, the purpose of this study was to identify at which point fatigue on neuromuscular parameters occurs in the knee flexors during a repeated sprint protocol as evaluated by decreases in PT and RTD measured in both limbs. We hypothesized that both sprint performance and neuromuscular parameters would significantly decrease after approximately 4–6 sprints.

## Methods

### Study design

This Cross-Sectional study was performed at Centro de Alto Rendimento Jamor between May/June 2021. The Ethical Committee at the Faculty of Human Kinetics at the University of Lisbon approved the study (#5/2021). All participants read and signed an informed consent form. All methods were performed in accordance with the Declaration of Helsinki.This study is reported following the STROBE guidelines [ref: https://www.strobe-statement.org/].

### Participants

We recruited healthy male participants aged between 18 and 30 who performed strength and conditioning training at least 4 times a week to participate in an indoor running track since they met the criteria of the Ethics Committee to participate in the present study. Participants were recruited by word of mouth locally in the university environment and at health clubs, and using social networks (e.g. Facebook and Instagram). The inclusion criteria were that participants had to be familiarized with exposure to maximal sprint bouts and maximal strength exercises, which in our university setting were only met by males. The exclusion criteria for potential participants were any history of: (i) serious knee or hip injuries in the past year (e.g., anterior cruciate ligament tear, medial collateral ligament, femoroacetabular shock, groin injuries that required surgery, and/or other serious injuries) in order to avoid compromising sprint performance and maximal voluntary isometric contraction (MVIC); (ii) previous hamstring strain injury in the last 2 years; (iii) lower back complaints or current complaints in the same region.

### Procedures

**Fig. 1:**
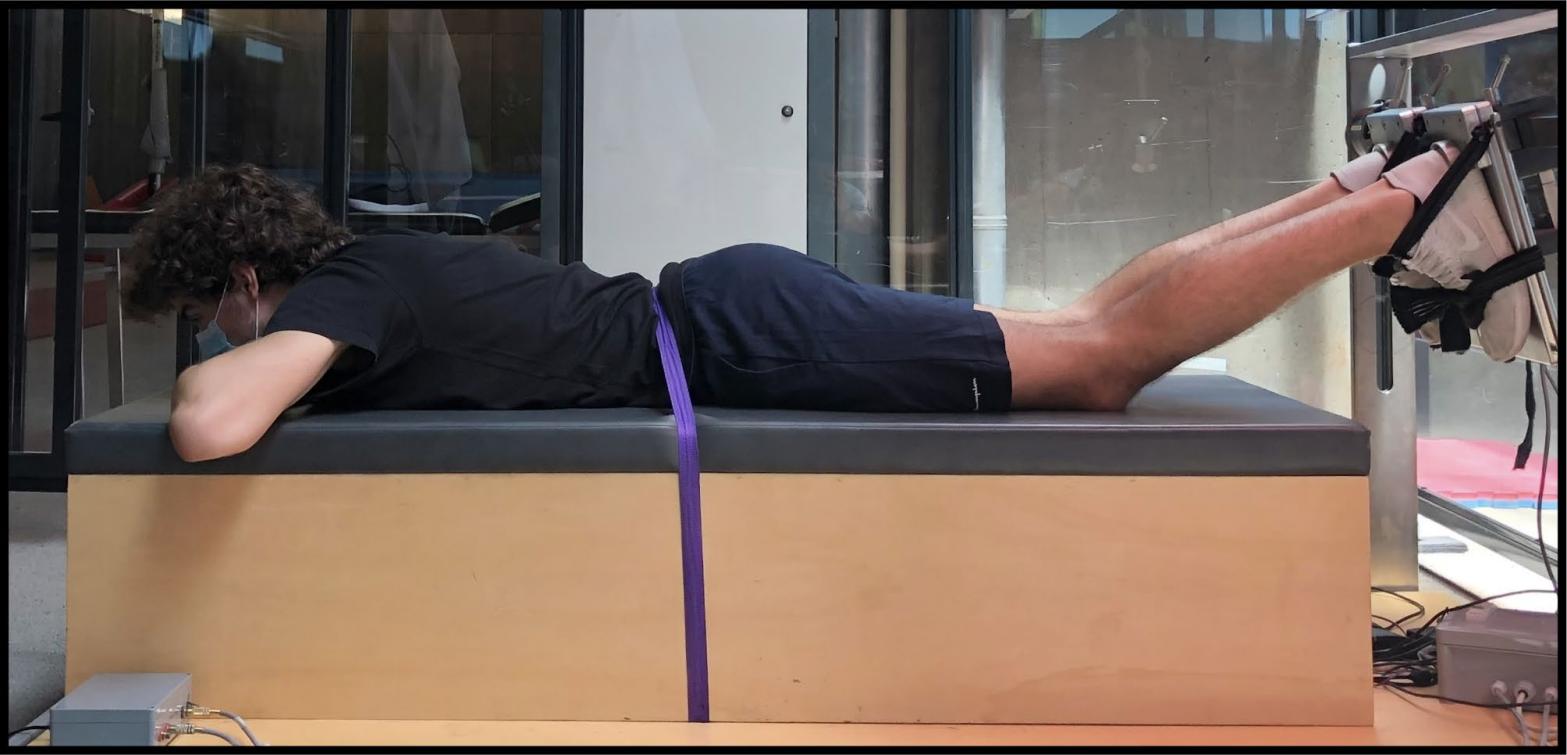
Experimental setup used to assess the knee flexors*’* peak torque and rate of torque development with 30° of knee flexion and neutral hip

Participants were placed in the prone position, with the hips in neutral anatomical position, knees flexed at 30º (0º = full extension) with the ankle at 15º plantar flexion, all the considerations for data acquisition were previously described [19]. Both feet were fixed in a foot holder containing a force transducer (Fig. 1) (Model STC, Vishay Precision, Malvern, PA, USA) at the heel level to collect the linear force perpendicular to the leg orientation and with the ankle at 90º [19]. Force data were amplified (Model UA73.202, Sensor Techniques, Cow- bridge, UK), digitally converted (USB-230 Series, Measurement Computing Corporation Norton, MA, USA), recorded using the DAQami software (v4.1, Measurement Computing Corporation, Norton, MA, USA). To estimate the knee torque, force data were multiplied by the perpendicular distance between the force transducer center and the femoral lateral condyle. Individuals were guided by visual feedback of force production during the assessments.

The neuromuscular parameters were determined using automated MATLAB routines (The Mathworks Inc., Natick, MA). The onset of force production was defined by visual inspection and recurring to mathematical models. These models were only applied to select a new onset if the instant where the torque value reached three standard deviations above the baseline value was different to the visual inspection. Average Sprint Speed (AvSS) was recorded using four photocells (Chronojump-Boscosystem, Spain) and data were processed using Chronojump software version 2.1.1–16.

### Protocol

Participants performed an indoor repeated sprint protocol, so wind had no effect on performance. The session started with familiarization for force production (i.e., 20 submaximal contractions) followed by two 3-s MVICs with a 30-s recovery period between trials. After a standardized warm-up protocol, a repeated maximal sprint task with five sets of two 30-m sprints (totalling 10 × 30 m) with space to decelerate afterwards was performed. To assess fatigue on neuromuscular parameters, participants performed two knee flexor MVICs with both limbs simultaneously after every two sprints to detected at which point fatigue on neuromuscular parameters occurs (with an equal and comparable measure), totaling 10 sprints and 12 MVICs performed “as fast and hard as possible”, to obtain both PT and RTD [20]. To ensure similar fatigue effects, time differences were measured and compared between participants such as the time between finishing the sprint and performing the first MVIC across the five intervals as well as the time between finishing a set and starting the next set. The participants were guided by a Strength and Conditioning Coach and Sports Scientist using feedback motivational to achieve the maximal sprint speed and MVIC in each set.

### Statistical analyses

Sample size was estimated using G*Power software (Heinrich-Heine Universität, Dusseldorf, Germany) for ANOVA repeated measures, considering a small effect size of 0.25, significance level of 0.05 and statistical power of 0.80. Data are presented as mean ± standard deviation. Data analysis was performed using IBM SPSS Statistics 27.0 (IBM Corporation, Armonk, NY). Normality of data distribution was confirmed using the Shapiro–Wilk test. Changes in sprint performance were examined by a one-way repeated measures ANOVA for the AvSS. The effect of recovery time on sprint performance was examined using a one-way repeated measures ANOVA for the time spent between finishing a set and starting the MVIC, and between finishing a set and starting the next set. Effects of the sprint task on neuromuscular parameters was examined using a two-way repeated measures ANOVA [sprint (before (0) and after 2, 4, 6, 8 and 10 sprints) 𝗑 limb (right and left)]. Statistical significance was set at *p* ≤ 0.05. Post-hoc analysis was conducted using Bonferroni correction.

## Results

Twenty resistance-trained male adults who perform strength and conditioning training at least four times per week participated in an indoor running track (age: 24.6 ± 5.0 years; height: 176.8 ± 6.5 cm; body mass: 77.3 ± 11.8 kg).

### Sprint performance

**Fig. 2:**
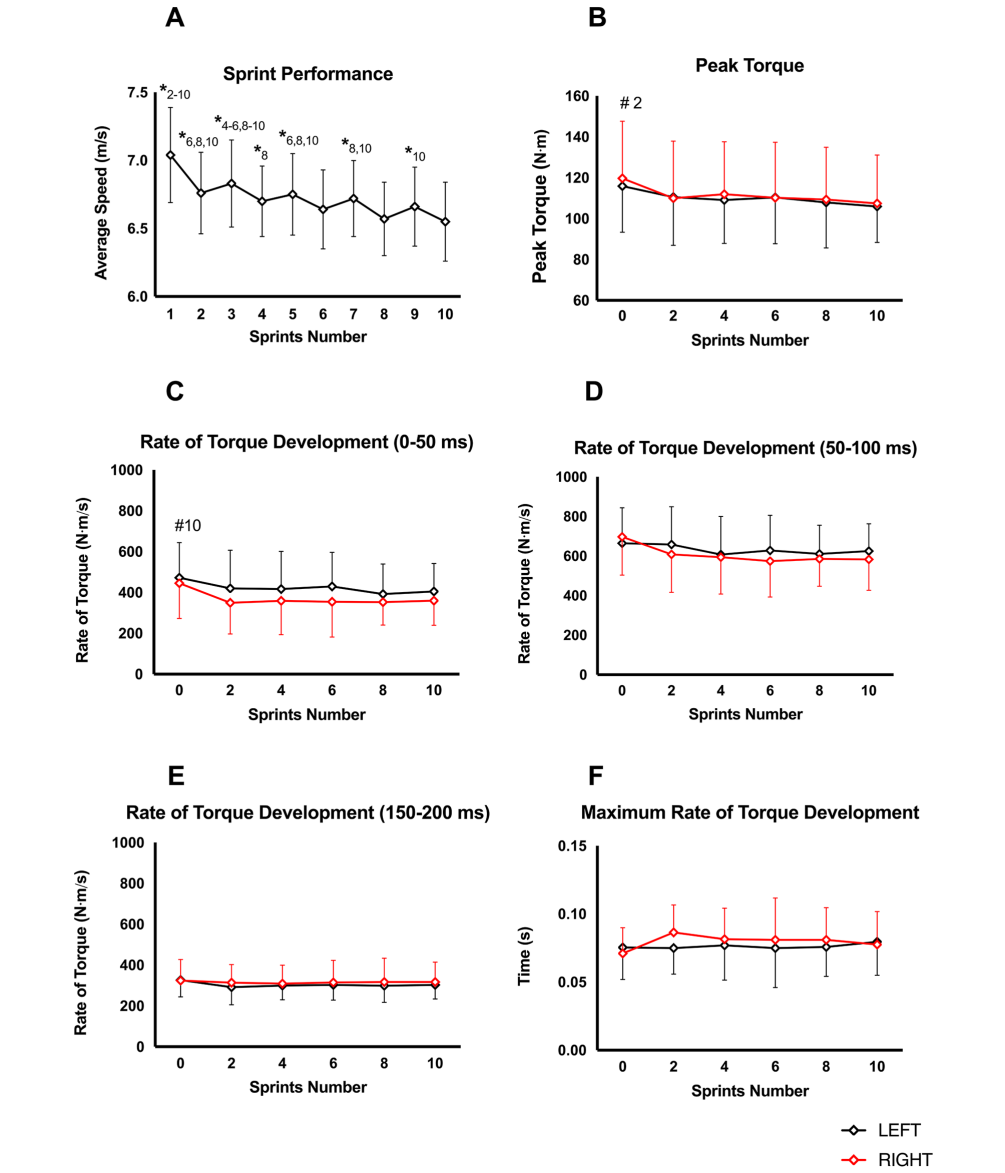
**(A)** Changes in average sprint speed (AvSS) during the repeated sprint protocol; **(B)** Changes in peak torque of both limbs; **(C-E)** Changes in rate torque of torque development in both limbs measured at 0-50ms **(C)** 50-100ms **(D)** 150-200ms **(E)**; **(F)** Changes in maximum rate of torque development. Black line corresponds to the left limb while the red line corresponds to the right limb. Data are presented as mean ± standard deviation *Significant difference between the current sprint and each subsequent sprint indicated (*p* ≤ 0.05) # Significant differences between instants (*p* ≤ 0.05)

No significant differences were observed in the time between finishing the sprint and performing the first MVIC across the five intervals (46.3 ± 4.7s; *p* = 0.423) or in the time between finishing a set and starting the next set (121.2 ± 7.6s; *p* = 0.503). There was a significant main effect of sprint number on AvSS (*p* < 0.001; η^2^_p_ = 0.643), which decreased by 6.9% (Fig. 2A) between the fastest (first) and slowest (last) sprint (first: 7.0 ± 0.4 m/s; last: 6.6 ± 0.3 m/s; *p* < 0.001).

### Neuromuscular parameters

The neuromuscular parameters results can be visualized in Fig. 2B-F. Regarding all neuromuscular parameters, PT demonstrated a significant main effect was found for the sprint number factor (*p* = 0.013; η^2^_p_ = 0.20). Post-hoc testing revealed significant differences from baseline to after the second sprint (baseline: 117.8 ± 24.5 N⋅m; after sprint 2: 110.2 ± 25.1 N⋅m; *p* = 0.020, *d* = 0.31). Moreover, a significant difference for instant (*p* = 0.008; η^2^_p_ = 0.149) was found for RTD 0-50ms. Post-hoc testing showed differences between before and after sprint 10 (baseline: 458.8 ± 162.3 N⋅m/s; after sprint 10: 382.6 ± 120.2 N⋅m/s). A significant effect was also found on RTD 150-200ms for instant (*p* = 0.015; η^2^_p_ = 0.135) however, post-hoc testing demonstrated no significant differences between all instants.

## Discussion

To the best of our knowledge, this is the first study to examine knee flexors’ neuromuscular parameters at multiple points (i.e., after every two sprints) during repeated sprinting. The main findings were (i) that AvSS decreased immediately after the first sprint; however, this decrease was only consistent from the first to the second sprint of each set; (ii) five sets of two sprints only led to a significant decrease in PT after the first two sprints and on RTD 0–50 ms after 10 sprints.

Contrary to our initial hypothesis, the AvSS decreased immediately after the first sprint; however, this decrease was only consistent from the first to the second sprint of each set. A possible explanation for the intermittent increase and decrease in AvSS can be explained by the 2 min of rest between sets (due to the experimental protocol design), as the first sprint of each set was always better than the second sprint of the previous set. Indeed, the importance of recovery time has previously been reported, since the absence of recovery time leads to impaired performance, as in the case of repeated-effort sprints [21]. Moreover, Balsom et al. (1992) reported (using these same rest periods) that longer periods (120s) could minimize metabolic muscle depletion and fatigue from first to last sprint compared to shorter periods (30s), indicating that the capacity to initially accelerate is restored rapidly during intervening recovery periods [22]. One possible explanation could be related to energy substrate availability. Increasing the recovery time allows for a greater restoration of phosphocreatine (PCr) stores [23, 24] and a greater contribution of anaerobic glycolysis to the production of adenosine triphosphate (ATP) which are both suggested to improve sprint performance [25, 26]. These factors explain why the first sprint of each set, which had a higher recovery time, had higher AvSS than the second one of the previous set.

Contrary to our initial hypothesis, the present study showed a significant difference in PT only after two sprints and on RTD 0–50 ms after 10 sprints. It should be noted that the development of fatigue on neuromuscular parameters (but not peak power output) has been reported to be unaffected by lower recovery periods of 10s compared to 30s [3], and 30s compared to 180s [27]; in the present study, the average recovery period before testing the neuromuscular parameters was 46.3 ± 4.7s. Therefore, while shorter recovery periods (30s) appear to cause greater decrements in sprint performance during repeated-sprint exercise, they do not seem to affect fatigue on neuromuscular parameters (46s) in the same way, although this could be task-specific [27]. It should be noted that sprinting is a more complex task (more degrees of freedom) compared to the mechanical task; in an isometric condition no other muscles than the knee flexors would contribute to the torque generated. Thus, as the knee flexors were not submitted to a significant degree of fatigue on neuromuscular parameters, the same cannot be inferred about the fatigue on neuromuscular parameters of other muscles associated with the sprint task, as the final outcome (i.e. AvSS) will be mediated by the action of various muscle groups (especially quadriceps). Moreover, fatigue on neuromuscular parameters has also been shown to be minimal 2 min after the end of a sprint protocol [4, 28, 29]. Thus, possibly repeated sprint exercise with ~ 2 min of recovery time interspersed between sprints (in our study) could reduce the development of fatigue on neuromuscular parameters.

Indeed, the later phase of the torque-time phase (> 75ms) is more correlated with MVIC and the speed related properties of the muscle inherent cross-bridge cycle and Ca^2+^ release [20]. Regarding the lower RTD 0–50 ms found following 10 sprints, this time interval (50ms) is within the early torque-time phase (< 75ms) which can be suggested that neural factors play a preponderant role since RTD at early-phase is dependent on neural factors such as motor unit discharge, lowered motor unit recruitment thresholds and increase spinal excitability [8, 20]. It should be noted that, for instance, via feedback from group III and IV afferents, metabolic changes have repeatedly been shown to decrease neural activation, thus suggesting a link between neural activation and metabolic changes and consequently recovery time [30]. This was only verified in the final set of sprints, where there is a higher accumulation of metabolites, potentially being the reason for the decrease. Besides that, we performed an analysis for the same individuals using a 10 × 30 m with 30 s interval sprints protocol (only measuring neuromuscular parameters before and after the task), verifying a greater effect size for the decrease in the early-phase RTD, especially RTD 0–50 ms (present study: η^2^_p_ = 0.149; previous study: η^2^_p_ = 0.222), and between RTD 50–100 ms (previous study: η^2^_p_ = 0.254) [31]. Indeed, it has been reported that early phase hamstring RTD (0-100 ms) is an important determinant for sprint acceleration performance and has been strongly associated with maximal sprinting speed [32]. Maximal sprinting speed is also considered to be highly dependent on the ability to quickly apply propulsive force onto the ground, thus relying on a high RTD [33], which could be the reason for the different results between studies with the same individuals. It must be considered that the present protocol does not promote strenuous loss of average speed; rather, the participants decrease their speed gradually, which could be also a possible reason to not impact the mechanical parameter results.

Finally, it has been reported that strength (i.e. force during MVC) [34], voluntary activation [35], and muscle contractile properties [34] recover rapidly after short-duration exercise, indicating a small window of opportunity for measuring fatigue. For instance, significant recovery in skeletal muscle function has been shown to occur within the first 1–2 min following exercise [34]. Similar to peripheral fatigue, measures of central fatigue are affected if a short time-delay exists between the task and measurement. In fact, measurements of fatigue during or post-repeated sprints have demonstrated that fatigue is transient and must be measured as soon as possible at the end of sprint [3]. In the present study, the best experimental design approach to measure in a sprint track (not with cycling equipment) reflects a time of 46s to measure the first MVIC across the different sets of each two sprints, indicating a limited fatigue impact.

This study has some limitations. Firstly, the time between the sprints and measurements must be considered, although it was not possible to reduce it any further. Secondly, only resistance-trained male adults without hamstring strain injury were included; therefore, the results cannot be generalized, as is the case for females.

## Conclusion

The present study demonstrated that measuring knee flexors fatigue on neuromuscular parameters during five sets of two running sprints only led to a significant decrease in PT after the first two sprints and on RTD 0–50 ms after 10 sprints. Coaches and sports science professionals should consider the present data to induce fatigue on the hamstring muscles using different time recovery and volume of sprints since a lower impact was seen with this time of recovery. Moreover, researchers should consider the present results when the goal is to assess the fatigue of knee flexors during repeated sprints since these results demonstrate limited impact to identify at which point fatigue on neuromuscular parameters occurs when evaluating PT and RTD every 2 sprints, due to small window of opportunity to measure fatigue on neuromuscular parameters. Therefore, future studies should create a protocol or a device that could measure the MVCs with intervals below 46 s for the first MVC and below 121 s between sprint sets as time of recovery is paramount to not detect major differences in the neuromuscular parameters. However, it could be extremely difficult to assess fatigue on neuromuscular parameters at multiple points for the same sprint session due to set up preparation since participants have to change the position (between running to knee flexion measurement). Finally, it seems that a time of 121s was sufficient for the recovery of performance, which created an intermittent decreasing shape in the sprint graph leading to a non continuous fatigue impact.

## Data Availability

The datasets generated during and/or analyzed during the current study are available from the corresponding author on reasonable request.
